# Therapeutic Biomarkers in Friedreich’s Ataxia: a Systematic Review and Meta-analysis

**DOI:** 10.1007/s12311-023-01621-6

**Published:** 2023-10-27

**Authors:** Maria Gavriilaki, Evangelia Chatzikyriakou, Maria Moschou, Marianthi Arnaoutoglou, Ioanna Sakellari, Vasilios K. Kimiskidis

**Affiliations:** 1 1st Department of Neurology, AHEPA University Hospital, School of Medicine, Aristotle University of Thessaloniki, 54124 Thessaloniki, Greece; 2 Laboratory of Clinical Neurophysiology, AHEPA University Hospital, School of Medicine, Aristotle University of Thessaloniki, Thessaloniki, Greece; 3 Hematology Department, Hematopoietic Cell Transplantation Unit, Gene and Cell Therapy Center, “George Papanikolaou” Hospital, Thessaloniki, Greece

**Keywords:** Friedreich’s ataxia, Therapeutics, Biomarker, Treatment outcome

## Abstract

Although a large array of biomarkers have been investigated in Friedreich’s ataxia (FRDA) trials, the optimal biomarker for assessing disease progression or therapeutic benefit has yet to be identified. We searched PubMed, MEDLINE, and EMBASE databases up to June 2023 for any original study (with ≥ 5 participants and ≥ 2 months’ follow-up) reporting the effect of therapeutic interventions on any clinical, cardiac, biochemical, patient-reported outcome measures, imaging, or neurophysiologic biomarker. We also explored the biomarkers’ ability to detect subtle disease progression in untreated patients. The pooled standardized mean difference (SMD) was calculated using a random-effects model. The study’s protocol was registered in PROSPERO (CRD42022319196). In total, 43 studies with 1409 FRDA patients were included in the qualitative synthesis. A statistically significant improvement was observed in Friedreich Ataxia Rating Scale scores [combining Friedreich Ataxia Rating Scale (FARS) and modified FARS (mFARS): SMD =  − 0.32 (− 0.62 to − 0.02)] following drugs that augment mitochondrial function in a sensitivity analysis. Left ventricular mass index (LVMI) was improved significantly [SMD =  − 0.34 (− 0.5 to − 0.18)] after 28.5 months of treatment with drugs that augment mitochondrial function. However, LVMI remained stable [SMD = 0.05 (− 0.3 to 0.41)] in untreated patients after 6-month follow-up. None of the remaining biomarkers changed significantly following any treatment intervention nor during the natural disease progression. Nevertheless, clinical implications of these results should be interpreted with caution because of low to very low quality of evidence. Further randomized controlled trials of at least 24 months’ duration using a biomarker toolbox rather than a single biomarker are warranted.

**Supplementary Information:**

The online version contains supplementary material available at 10.1007/s12311-023-01621-6.

## Background

Friedreich’s ataxia (FRDA) is the most common inherited ataxia affecting about 1 in 50,000 Europeans [1]. It is a rare hereditary autosomal recessive disease caused by mutations in the frataxin (FXN) gene resulting in decreased levels of functional frataxin protein [2]. The exact pathophysiologic repercussions of FXN loss are not yet fully understood. FXN is involved in mitochondrial iron homeostasis through iron-sulfur cluster synthesis and iron storage regulation [3]. FRDA is a progressive multisystem disorder presenting with limb ataxia, proprioception loss, gait and speech disturbances, myocardial involvement, and skeletal deformities with typical age of onset during puberty [4]. Moreover, diabetes mellitus, visual deficits, and hearing loss often co-exist. During early adulthood, most FRDA patients are non-ambulatory [5]. However, myocardial involvement represents the major determinant of survival [6].

Recently, omaveloxolone, a potent activator of Nrf2 which represents a key mediator of the antioxidant response in FRDA but also a NF-kB-mediated inflammatory response suppressor, a trait with unclear effects on FRDA pathophysiology, was approved by the FDA as the first agent for the therapeutic management of adults and adolescents aged 16 years and older with FRDA [7, 8]. In general, therapeutic approaches can be categorized into three groups based on their mechanism of action. The mainstay in FRDA patients’ management relies on symptomatic approaches such as drugs for cardiac arrhythmias, cerebellar symptoms, and diabetes. The second group comprises drugs that augment mitochondrial function like omaveloxolone, idebenone, EPI-743, deferiprone, riboflavin, epicatechin, coenzyme Q10, vitamin E, l-carnitine, and creatine. The third group includes drugs that may possibly increase frataxin levels such as erythropoietin, interferon gamma, resveratrol, and nicotinamide. In addition, research efforts led to the emergence of potential disease-modifying FRDA treatments mainly based on frataxin gene modulation [9].

The difficulty in demonstrating possible efficacy of various agents tested in clinical trials could be attributed, to some degree, to the lack of a suitable quantifiable biomarker detecting slow disease progression or subtle response to a possibly effective treatment in the context of a trial’s timeline. This issue is commonly encountered in the design of trials involving patients with rare, slowly progressive heterogeneous neurodegenerative disorders [10, 11]. It should be a key priority to stratify FRDA subpopulations and utilize the most effective outcome measure for each subgroup that could depict subtle disease progression and thus responsiveness to various therapies [12]. The European Friedreich’s Ataxia Consortium for Translational Studies (EFACTS) study group recently examined 602 treatment-naive FRDA patients in an effort to provide sensitive outcome measures to monitor change over time in different stages of the disease [13].

The objective of this study is to systematically review the literature and conduct a meta-analysis to summarize and evaluate the biomarkers used to assess therapeutic efficacy in Friedreich’s ataxia patients receiving any treatment.

## Materials and Methods

Our protocol was pre-published online at the International Prospective Registry of Systematic Reviews (PROSPERO accession number CRD42022319196).

The reporting of this systematic review and meta-analysis follows the Preferred Reporting Items for Systematic Reviews and Meta-Analyses (PRISMA) 2020 statement (online-only Supplementary material 1) [14].

### Search Strategy and Eligibility Criteria

We searched MEDLINE (via PubMed), EMBASE (via Ovid), and the Cochrane Library for original studies (with more than five participants) investigating the effect of therapeutic interventions on any biomarker in patients with genetically confirmed FRDA. We excluded case reports which were defined as studies that included ≤ 4 patients based on previous literature reviews [15, 16]. We included published full-text articles that provided numerical analysis of biomarker changes measured at least during two different timepoints (with a minimum 2-month interval). The search strategy was developed based on the combination of the following key words: Friedreich’s Ataxia, therapeutics, and treatment outcome as presented at online-only Supplementary material 2. We also reviewed references of previous systematic reviews and included records. We did not apply any language or year of publication filter. We completed the literature search on March 18, 2022 and updated it on June 3, 2023.

### Data Extraction (Selection and Coding)

Title and abstract screening along with duplicate record removal were performed independently by two reviewers (M.G., M.M.). Two reviewers (M.G., E.C.) examined the full texts of the remaining records. Any disagreement regarding study eligibility was resolved by a senior author (V.K.). We extracted unadjusted raw data on a standardized Microsoft Excel spreadsheet regarding study characteristics, patients’ baseline characteristics, intervention type, the mean with corresponding SD, and 95% CI for each biomarker on two different time points. Whenever studies did not report mean and SD, we calculated the mean and SD from the data provided (sample size, IQR, SEM) [17]. For records that did not report quantitative assessments of some biomarkers, we extracted relative information to conduct a narrative summary of the main findings.

### Risk-of-Bias Assessment in Individual and across Studies

To ascertain the validity of the included records, two independent reviewers (M.G. and E.C.) assessed risk of bias (RoB) using the revised Cochrane Collaboration’s Risk of Bias tool for randomized controlled trials (RCTs), Newcastle–Ottawa Scale (NOS) for case–control or cohort studies, Risk Of Bias In Non-randomized Studies of Exposure (ROBINS-E) for open-label trials, and a tool proposed by Murad et al. for case series [18–21]. According to these scales, the studies were ranked as high, fair, or low risk of bias. We also aimed to evaluate the quality of evidence applying the Grading of Recommendations, Assessment, Development and Evaluations (GRADE) approach [22].

### Summary Measures and Synthesis of Results

We conducted a narrative summary of the records included in this systematic review, reporting the biomarkers examined and the therapeutic effect as reported in each study using descriptive statistics. First, biomarkers were grouped as follows: (a) clinical outcome measures, (b) cardiac biomarkers, (c) biochemical biomarkers, (d) patient-reported outcome measures (PROMs), (e) imaging biomarkers, (f) neurophysiologic biomarkers, and (g) other biomarkers. Second, records were classified according to the mechanism of action of the administered intervention into three groups: (1) drugs that augment mitochondrial function, (2) drugs that increase frataxin, and (3) symptomatic treatment. The assignment of each record retrieved from literature search in each group of therapeutic approaches according to the mechanism of action of the administered intervention was based on Friedreich’s Ataxia Research Alliance (FARA) treatment pipeline [23].

The primary outcome of interest was the change from baseline score of any biomarker examined following therapeutic intervention. We also aimed to investigate the change from baseline score of any biomarker in the placebo or no treatment group separately to evaluate the ability of the biomarker to detect subtle disease progression.

We performed a quantitative synthesis for each biomarker type whenever possible using Comprehensive Meta-analysis software (version 3.0; Biostat Inc.). We calculated the standardized mean difference (SMD) with a 95% confidence interval (CI) with a significance level set at *p* < 0.05 whenever three or more studies with the same intervention type reported the same biomarker. We calculated the effect size for each biomarker using a random-effect model based on follow-up sample size. We used a conservative value of 0.5 as a correlation between pre- and post-treatment assessments whenever this correlation was not reported in the original record [11]. Whenever data from the same cohort were published in more than one record, we included in the analysis the most relevant study with the largest sample size to eliminate overlapping samples. Publication bias and heterogeneity were examined by visual assessment of the funnel plots and by calculating the *I*^2^, respectively [24, 25].

We planned sensitivity analyses based on risk-of-bias assessment along with subgroup analysis according to drug administered. Additional explorative analyses were planned depending on data availability according to population age (children, adolescents, or adults) or different follow-up timepoints. However, there were insufficient data to perform these subgroup analyses.

## Results

### Descriptive Characteristics and RoB within Studies

**Fig. 1:**
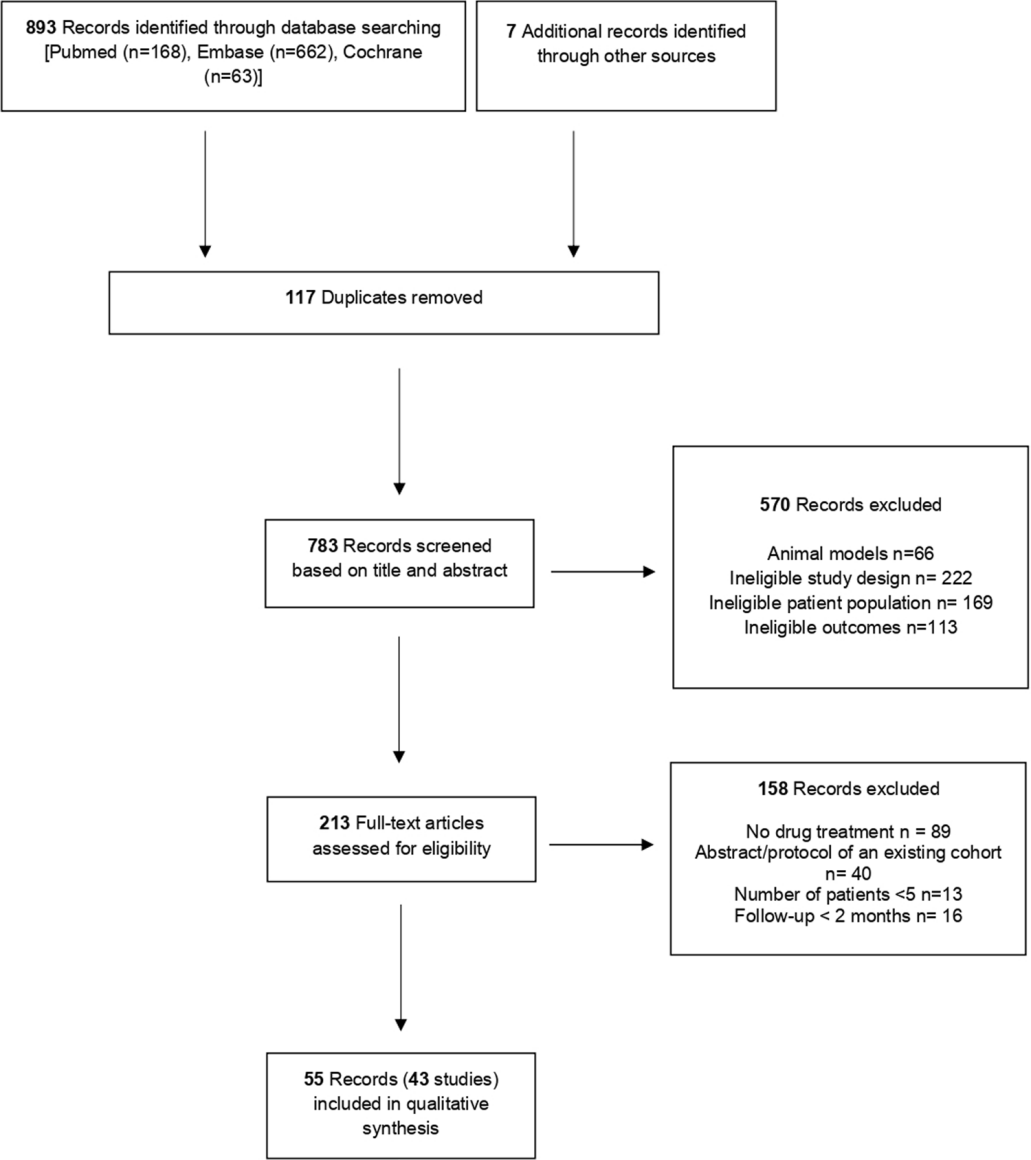
Flowchart of study selection

Among 783 unique records retrieved from the literature search, 55 records fulfilled the prespecified inclusion criteria. Study selection process is presented as a PRISMA flow diagram in Fig. 1.

We included 43 studies presented at 55 records in the final qualitative synthesis. These 43 studies examined 1409 FRDA participants (age range 4–74 years) during a follow-up period of 2 to 132 months. The intervention type included nine different drugs or drug combinations that augment mitochondrial function in 25 studies (31 records), four drugs or drug combinations that increase frataxin in ten studies (16 records), or eight different symptomatic treatments in eight studies/records. The 55 records included 19 RCTs, 25 open-label trials, 10 cohort studies, and one case series. The characteristics of the included records are summarized in Table 1.

**Table 1:** Descriptive characteristics of 53 studies included in the qualitative synthesis

Study’s ID	Record	Study type	Drug	Biomarker type	*N*	Age	Follow-up*	RoB
Drugs that augment mitochondrial function								
Arpa 2014 [26]		Open-label trial	Deferiprone, idebenone, riboflavin	Clinical, cardiac, PROMs	13	14–61	45	High
Artuch 2002 [27]		Open-label trial	Idebenone	Clinical, neurophysiologic, cardiac	9	11–19	12	High
Boddaert 2007 [28]		Phase I–II trial	Deferiprone	Clinical, imaging	13	14–23	6	High
Brandsema 2010 [29]		Prospective cohort	Idebenone	Clinical, PROMs	7	13–19	12	High
Buyse 2003 [30]		Prospective cohort	Idebenone	Clinical, biochemical, cardiac	8	8.6–27.1	12	High
Cook 2019 [31]		RCT	Idebenone	Clinical, PROMs	29	15–73	2	Low
UCL cohort	Cooper 2008 [32]	RCT	Q10 and vitamin E	Clinical, cardiac, PROMs	50	10.6–58.5	24	Low
	Hart 2005 [33]	Open-label trial	Q10 and vitamin E	Clinical, cardiac, imaging	10	10–57.7	47	High
	Lodi 2001 [34]	Open-label trial	Q10 and vitamin E	Clinical, cardiac	10	16–40	6	High
NINDS cohort	Di Prospero 2007 [35]	RCT	Idebenone	Clinical, biochemical, PROMs	48	9–17	6	Low
	Drinkard 2010 [36]	RCT	Idebenone	Cardiac, other	48	9–17	6	Fair
Elincx-benizri 2016 [37]		Case series	Deferiprone and idebenone	Clinical, PROMs, cardiac	7	16–36	24	Low
Hausse 2002 [38]		Prospective cohort	Idebenone	Cardiac	38	4–22	6	High
MOXIe Study	Lynch 2021 [39]	RCT	Omaveloxolone	Clinical, cardiac, PROMs	103	16–40	12	Low
	Lynch 2018 [8]	Phase II, RCT	Omaveloxolone	Clinical, PROMs, other	69	16–37	3	Low
	Lynch 2022 [40]	Open-label extension trial	Omaveloxolone	Clinical	73	16–40	36	High
IONIA study	Lynch 2010 [41]	RCT	Idebenone	Clinical, PROMs	70	8–18	6	Low
	Meier 2012 [42]	Open-label extension trial	Idebenone	Clinical	70	8.5–18.6	18	High
Mariotti 2003 [43]		RCT	Idebenone	Clinical, cardiac	29	20.8–31.8	12	High
Pandolfo 2014 [44]		RCT	Deferiprone	Clinical, cardiac, PROMs	72	7–35	6	Fair
Paredes-Fuentes 2021 [45]		Retrospective cohort	Idebenone	Clinical, cardiac	27	7–21	132	High
Pineda 2008 [46]		Prospective cohort	Idebenone	Clinical, cardiac, biochemical	24	8–46	60	High
Qureshi 2020 [47]		Open-label trial	Epicatechin	Clinical, cardiac, imaging, biochemical, PROMs	10	10–22	6	High
Ribaï 2007 [48]		Open-label trial	Idebenone	Clinical, cardiac	113	13–74	60	High
Rinaldi 2009 [49]		Retrospective cohort	Idebenone	Clinical, cardiac	35	26.9 ± 14.9	60	High
Rustin 2002 [50]		Retrospective cohort	Idebenone	Cardiac	40	4–11	6	High
Schöls 2005 [51]		RCT	l-Carnitine and creatine	Clinical, cardiac, imaging	54	15–63	4	Fair
Schulz 2000 [52]		Open-label trial	Idebenone	Biochemical	8	na	2	High
Sival 2009 [53]		Retrospective cohort	Idebenone	Clinical, neurophysiologic	6	6–18	24	High
Velasco-Sánchez 2011 [54]		Prospective cohort	Deferiprone and idebenone	Clinical, cardiac, imaging	20	8–25	11	High
Zesiewicz2018 [55]		RCT	EPI-743	Clinical, cardiac	63	28.7 ± 6.0	24	High
Drugs that increase frataxin								
Innsbruck cohort	Boesch 2008 [56]	Open-label trial	RhuEPO	Clinical, biochemical, PROMs	8	26–55	6	Low
	Santner 2014 [57]	Open-label trial	RhuEPO	Imaging	21	18–46	6	High
	Egger 2013 [58]	Open-label trial	RhuEPO	Clinical, imaging	12	18–51	12	High
Libri 2014 [59]		Open-label trial	Nicotinamide	Clinical, biochemical, PROMs	10	19–54	2	High
Lynch 2019 [60]		RCT	IFNγ-1b	Clinical, biochemical, PROMs	92	10–25	6	Low
Mariotti 2012 [61]		RCT	RhuEPO	Clinical, biochemical, PROMs	16	18–40	6	High
Austrian cohort	Nachbauer 2011 [62]	Open-label trial	RhuEPO	Clinical, biochemical	5	49 (IQR 31–52)	3	High
	Nachbauer 2011 [63]	Open-label trial	RhuEPO	Clinical, biochemical	7	40 ± 14	2	High
	Nachbauer 2012 [64]	Open-label trial	RhuEPO	Other, biochemical	11	40 ± 14	2	High
	Nachbauer 2013 [65]	Open-label trial	RhuEPO	Imaging	15	40 ± 14	2	Low
Saccà 2016 [66]		RCT	EPO	Clinical, biochemical, cardiac, PROMs	56	35.4 ± 13.1	12	Fair
Saccà 2011 [67]		Open-label trial	EPO	Clinical, cardiac, biochemical biomarkers	10	29 ± 8.2	15	High
Seyer 2014 [68]		Open-label trial	IFNγ-1b	Clinical, biochemical biomarkers, PROMs	12	8–17	3	Low
IRCCS cohort	Vavla 2020 [69]	Open-label trial	IFNγ-1b	Clinical, biochemical, cardiac	12	11–26	18	High
	Vavla 2020 [70]	Open-label trial	IFNγ-1b	Imaging	12	11–26	18	High
Yiu 2015 [71]		Open-label, non-randomized trial	Resveratrol	Clinical, biochemical, PROMs, cardiac	27	> 18	3	High
Symptomatic treatment								
Botez 1996 [72]		RCT	Amantadine hydrochloride	Clinical	28	19–47	4	High
Casazza 1986 [73]		Open-label trial	Verapamil	Cardiac	47	10–34	24	High
Costantini 2016 [74]		Prospective cohort	Thiamine	Clinical, biochemical, cardiac, PROMs	34	36.3 ± 11.1	24	High
Naeije 2023 [75]		RCT	ctDCS	Clinical, imaging	24	15–66	3	High
Patel 2019 [76]		Open-label trial	Methylprednisolone	Clinical, PROMs, biochemical	11	9–65	6	High
Sanz-Gallego 2014 [77]		Open-label trial	Insulin/IGF-1	Clinical, cardiac, PROMs	5	23–36	36	Fair
Trouillas 1995 [78]		RCT	5-Hydroxytryptophan levorotatory form	Clinical	26	28.5 ± 9.4	6	Fair
Wang 2021 [79]		RCT	Luvadaxistat	Clinical, PROMs	67	18–55	3	High

*N* number of patients, *RoB* risk of bias, *PROMs* patient-reported outcome measures, *RCT* randomized controlled trial, *ctDCS* cerebellar transcranial direct current stimulation, *RhuEPO* recombinant human erythropoietin, *EPO* erythropoietin, *IFNγ-1b* interferon gamma-1b, *IGF-1* insulin-like growth factor 1, *UCL* University College London, *NINDSL* National Institute of Neurological Disorders and Stroke, *IRCCS* Istituto di Ricovero e Cura a Carattere Scientifico

*Maximum follow-up period presented in months

Eleven records were deemed of low risk of bias (RoB), six of fair quality, and 38 were deemed of high RoB. The majority of non-randomized studies (31 out of 36 open-label, cohort, or case-series) presented high risk of bias mainly because of the inadequate identification of potential confounding factors. Only seven RCTs had high RoB.

### Clinical Outcome Measures

The effect of drugs that augment mitochondrial function on clinical outcome measures was examined in 22 studies [26] [27–31, 35, 37] [8, 32, 39–42, 44–49, 51, 53–55] [43]. Ten studies investigated clinical outcome measures following drugs that increase frataxin [56, 58–63, 65–69, 71] while seven studies following symptomatic treatment [72, 74, 76–79] [75]. The clinical scales examined along with treatment effect in each study are presented in Table 2.

**Table 2:** Clinical outcome measures examined in each study included in the qualitative synthesis

ID	Drug	Biomarker	*N*	Follow-up*	Clinical effect
Drugs that augment mitochondrial function					
Arpa 2014	Deferiprone, idebenone, riboflavin	SARA	13	45	Deterioration^+^
Artuch 2002	Idebenone	ICARS	9	12	Improvement^+^
Boddaert 2007	Deferiprone	ICARS, Perdue Pegboard test	13	6	Improvement
Brandsema 2010	Idebenone	ICARS	7	12	Deterioration
Buyse 2003	Idebenone	CAGRS	8	12	Deterioration
Cook 2019	Idebenone	ICARS, 9‐HPT, speech assessments, CGI‐C	29	2	Improvement for ambulant pts^+^
Di Prospero 2007	Idebenone	ICARS, FARS	48	6	Improvement for ambulant pts^+^
Elincx-benizri 2015	Deferiprone and idebenone	SARA, FARS	5	24	Inconclusive
IONIA study [41]	Idebenone	ICARS, FARS, FACT-Z3	68	18	Improvement
MOXIe Study [8, 39, 40]	Omaveloxolone	mFARS, T25FW, 9-HPT, LCLA	149	36	Improvement^+^ (93% ambulant pts)
Mariotti 2003	Idebenone	ICARS	29	12	No difference
Pandolfo 2014	Deferiprone	ICARS, FARS, 9-HPT, T25FW, LCLA	72	6	Inconclusive
Paredes-Fuentes 2021	Idebenone	ICARS	18	132	Deterioration^+^
Pineda 2008	Idebenone	ICARS	24	60	Deterioration in adults^+^
Qureshi 2020	Epicatechin	FARS, 9-HPT, 8-m timed walk	10	6	Improvement
Ribaï 2007	Idebenone	ICARS, oculomotor function, writing test	104	84	Deterioration
Rinaldi 2009	Idebenone	IACRS	35	60	Deterioration
Schöls 2005	l-Carnitine and creatine	ICARS	54	4	No difference
Sival 2009	Idebenone	ICARS	6	24	Deterioration^+^
UCL cohort [32]	Q10 and vitamin E	ICARS	50	24	Deterioration
Velasco-Sánchez 2011	Deferiprone and idebenone	ICARS	19	11	No difference
Zesiewic 2018	EPI-743	FARS, 9-HPT, T25FW, LCLA	63	24	Improvement^+^
Drugs that increase frataxin					
Innsbruck cohort [56, 58]	RhuEPO	SARA, FARS, 9-HPT	9	8	Improvement^+^
Libri 2014	Nicotinamide	SARA, SCAFI, SIT	10	2	No difference
Lynch 2019	IFNγ-1b	mFARS, FARS, T25FW, 9-HPT, LCSLC	92	6	No difference
Mariotti 2012	RhuEPO	SARA, 9-HPT	16	6	No difference
Nachbauer 2011	RhuEPO	SARA	7	2	Inconclusive
Saccà 2011	EPO	ICARS	10	9	No difference
Saccà 2016	EPO	SARA, 9-HPT	56	12	Improvement^+^
Seyer 2014	IFNγ-1b	FARS, T25FW, 9-HPT	10	3	Improvement^+^
Vavla 2020	IFNγ-1b	SARA	12	18	Improvement
Yiu 2015	Resveratrol	FARS, SARA, ICARS, speech and audiologic function	24	3	Improvement^+^
Symptomatic treatment					
Botez 2008	Amantadine hydrochloride	Simple visual and auditory reaction time and movement time	28	4	No difference
Costantini 2016	Thiamine	SARA, Archimedes’ spiral	34	24	Improvement^+^
Naeije 2023	ctDCS	SARA, CCFS, CCAS-S	24	3	Improvement^+^
Patel 2019	Methylprednisolone	T25FW, 1 MW, FARS, 9-HPT	11	6	Improvement
Sanz-Gallego 2014	Insulin/IGF-1	SARA	5	12	Inconclusive
Trouillas 1995	5-Hydroxytryptophan levorotatory form	Quantitative measurements of time evaluating stance, speech, writing, and drawing	19	6	Improvement
Wang 2021	Luvadaxistat	9-HPT^−1^, mFARS, T25FW, LCSLC	67	3	No difference

*1MW* 1-min walk; *ctDCS* cerebellar transcranial direct current stimulation; *CAGRS* Cooperative Ataxia Group Rating Scale; *CCFS* composite cerebellar functional severity score; *CCAS-S* Cerebellar Cognitive Affective Syndrome Scale; *CGI‐C* Clinical Global Impression of Change; *ICARS* International Cooperative Ataxia Rating Scale; *IACRS* Inherited Ataxia Clinical Rating Scale; *FARS* Friedreich Ataxia Rating Scale; *SARA* Scale for the Assessment and Rating of Ataxia; *T25FW* Timed 25-Foot Walk; *9-HPT* 9-hole peg test; *FACT-Z3* Friedreich’s Ataxia Composite Test derived from the Timed 25-Foot Walk test, the 9-hole peg test, and the Low-Contrast Letter Acuity test; *LCLA* Low-Contrast Letter Acuity; *SIT* Speech Intelligibility Test; *SCAFI* spinocerebellar ataxia functional index; *LCSLC* low-contrast Sloan letter chart; *RhuEPO* recombinant human erythropoietin; *EPO* erythropoietin; *IFNγ-1b* interferon gamma-1b; *IGF-1* insulin-like growth factor 1; *Pts* patients

*Maximum follow-up period presented in months

^+^Statistically significant

#### Drugs that Augment Mitochondrial Function

**Fig. 2:**
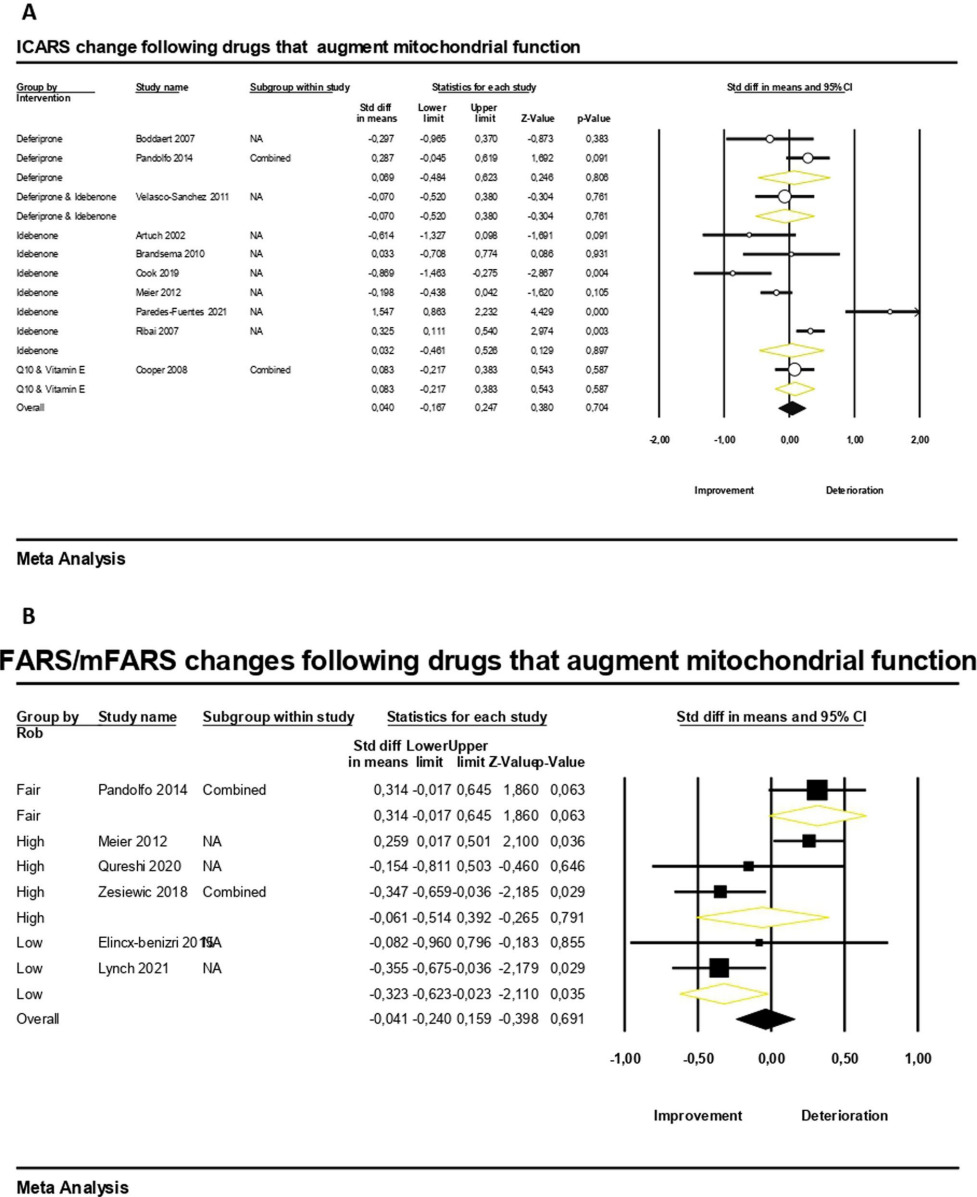
Clinical outcome measures changes (**A** ICARS, **B** Friedreich Ataxia Rating Scale scores (FARS/mFARS)) following drugs that augment mitochondrial function

We performed a pooled analysis of studies that reported on International Cooperative Ataxia Rating Scale (ICARS) and Friedreich Ataxia Rating Scale scores [including Friedreich Ataxia Rating Scale (FARS) and modified FARS (mFARS)] changes following an intervention targeting mitochondrial function as presented in Fig. 2.

The pooled mean effect size of ten studies examining ICARS showed no statistically significant changes after 12 months of treatment (SMD = 0.03, 95% CI − 0.26 to 0.32, *p* = 0.8, *I*^2^ = 80%, follow-up range 2–132 months), with substantial heterogeneity that was not eliminated even at the pre-planned sensitivity and subgroup analyses of studies grouped by drug administered (Fig. 2A). The results were similar when we pooled six studies reporting on Friedreich Ataxia Rating Scale scores (FARS or mFARS) changes following treatment with drugs that augment mitochondrial function (SMD =  − 0.05, 95% CI − 0.34 to 0.25, *p* = 0.8, *I*^2^ = 72%) during a follow-up period of 6 to 24 months (Fig. 2B). However, sensitivity analysis using studies of low RoB revealed a statistically significant improvement (SMD =  − 0.32, 95% CI − 0.62 to − 0.02, *p* = 0.04, *I*^2^ = 0%) on Friedreich Ataxia Rating Scale scores (FARS/mFARS) following drugs that augment mitochondrial function (Fig. 2B). Of note, no statistically significant improvement was found when we pooled five studies reporting on FARS changes alone following treatment with drugs that augment mitochondrial function (SMD = 0.39, 95% CI − 0.55 to 1.33, *p* = 0.42, *I*^2^ = 95%) suggesting that the positive result was mainly driven by the omaveloxolone study in which mFARS has been used as an outcome measure (online-only Supplementary material 3).

#### Drugs that Increase Frataxin

**Fig. 3:**
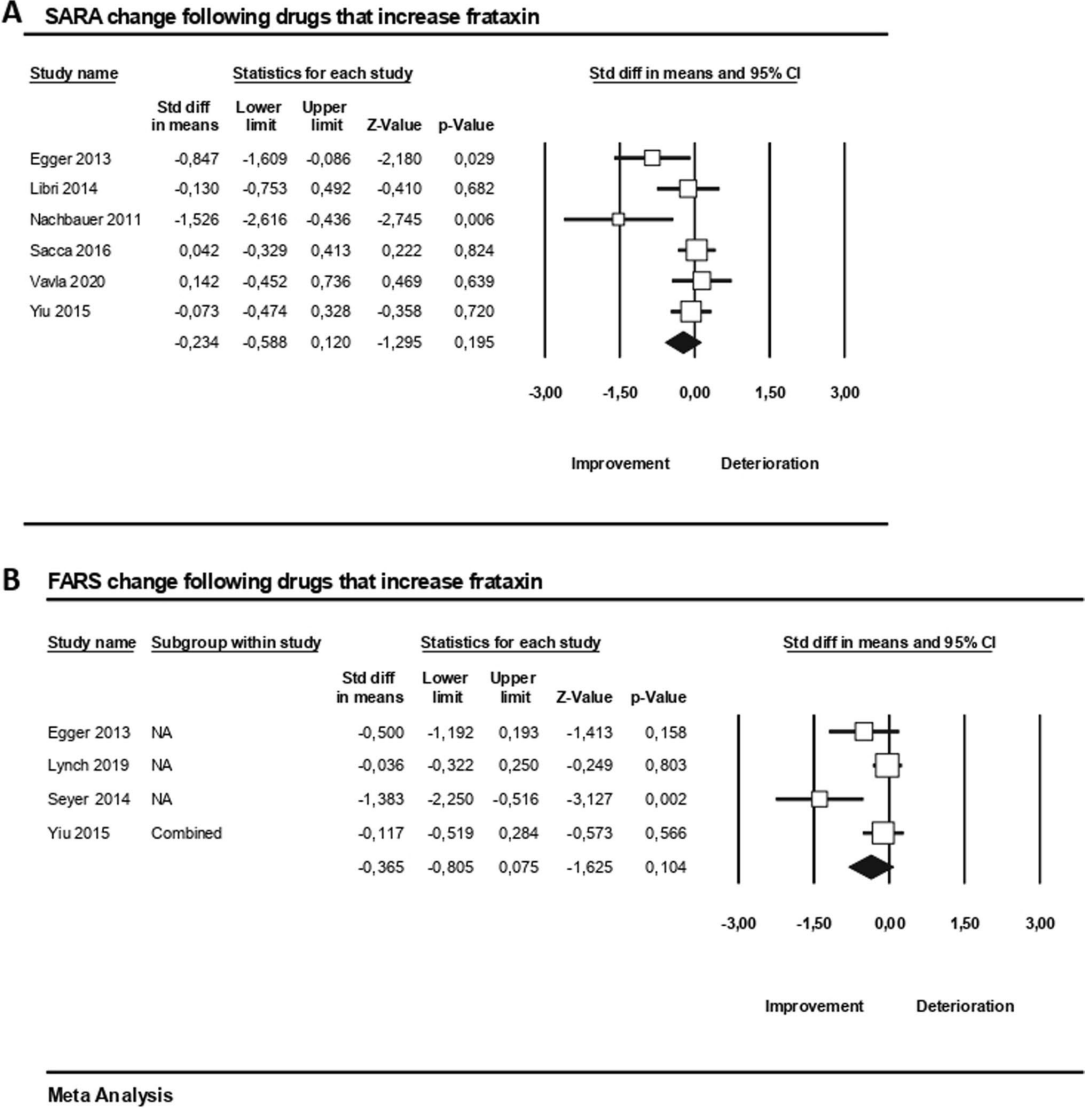
Clinical outcome measures changes (**A** SARA, **B** FARS) following drugs that increase frataxin

The Scale for the Assessment and Rating of Ataxia (SARA) was not affected by drugs that increase frataxin (SMD =  − 0.23, 95% CI − 0.59 to 0.12, *p* = 0.2, *I*^2^ = 56%) according to six studies included in the quantitative synthesis during a median follow-up period of 7.5 months (range 2–18) (Fig. 3A). Moreover, the pooled effect estimates showed no significant difference between pre- and post-drugs that increase frataxin on FARS based on four studies with a median follow-up of 3 months (SMD =  − 0.37, 95% CI − 0.81 to 0.08, *p* = 0.1, *I*^2^ = 67%, Fig. 3B).

#### Symptomatic Treatment

The heterogeneity of drugs used, follow-up periods along with the different outcome measures applied impeded a pooled synthesis of these seven studies (Table 2) [59, 61–65].

### Cardiac Biomarkers

Echocardiogram parameters were used as outcome measures in 27 studies [38] [50, 73]. Descriptive characteristics are presented in Table 3.

**Table 3:** Cardiac biomarkers examined in each study included in the qualitative synthesis

ID	Drug	Biomarker	*N*	Clinical effect
Drugs that augment mitochondrial function				
Arpa 2014	Deferiprone, idebenone, riboflavin	LVMI, LVEF	13	Stable
Artuch 2002	Idebenone	IVS, LVPW	9	Stable
Buyse 2003	Idebenone	LVMI, IVS, LVPW	8	Improvement^+^
Elincx-benizri 2015	Deferiprone and idebenone	IVS, LVPW, LVEF	4	Inconclusive
Hausse 2002	Idebenone	LVMI, IVS, FS	38	Improvement^+^
MOXIe Study[39]	Omaveloxolone	Echocardiogram	103	Stable
Mariotti 2003	Idebenone	IVS, LVPW, LVMI, LVEF	29	Improvement^+^
NINDS cohort [36]	Idebenone	LVEF	48	Stable
Pandolfo 2014	Deferiprone	LVEF, LVMI, FS	72	Improvement^+^
Paredes-Fuentes 2021	Idebenone	IVS, LVPW, LVMI, LVEF	27	Stable
Pineda 2008	Idebenone	FS, LVEF, IVS, LVPW, LVMI	24	Inconclusive
Qureshi 2020	Epicatechin	LVMI, LVEF, IVS, NT-Pro BNP, ST2, troponin	10	Deterioration^+^
Ribaï 2007	Idebenone	LVMI, LVEF, LVPW, FS, IVS	104	Inconclusive
Rinaldi 2009	Idebenone	LVEF, LVPW, IVS	35	Deterioration^+^
Rustin 2002	Idebenone	LVMI	40	Improvement^+^
Schöls 2005	l-Carnitine and creatine	IVS, LVPW, FS, LVMI	54	Stable
Sival 2009	Idebenone	IVS, LVPW, NT-pro BNP	6	Stable
UCL cohort [32]	Q10 and vitamin E	IVS, FS, LVPW	50	Improvement^+^
Velasco-Sánchez 2011	Deferiprone and idebenone	LVMI, LVEF, IVS, FS	20	Improvement^+^
Zesiewic 2018	EPI-743	Echocardiogram	63	Stable
Drugs that increase frataxin				
Saccà 2011	Epoetin alfa	Echocardiogram	10	Stable
Saccà 2016	Epoetin alfa	LVMI, LVEF	56	No difference
IRCCS cohort	IFNγ-1b	IVS, LVEF, LVED, FS, LVWT	12	Improvement
Yiu 2015	Resveratrol	LVED, LVMI, LVEF	24	Stable
Symptomatic treatment				
Casazza 1986	Verapamil	IVS, LVPW, LVED, LVMI	47	No difference
Costantini 2016	Thiamine	LVEF, IVS, LVPW	13	Improvement^+^
Sanz-Gallego 2014	IGF-1	IVS, LVPW, FS, LVEF, LVMI	5	Stable

*LVMI* left ventricular mass index, *LVEF* left ventricular ejection fraction, *IVS* intraventricular septal wall, *LVPW* left ventricular posterior wall, *LVED* left ventricular end-diastolic diameter, *LVWT* left ventricular wall thickness, *FS* fractional shortening, *NT-Pro BNP* serum N-terminal pro B-type natriuretic peptide, *ST2* suppressor of tumorigenicity 2

*Maximum follow-up period presented in months

^+^Statistically significant

#### Drugs that Augment Mitochondrial Function

**Fig. 4:**
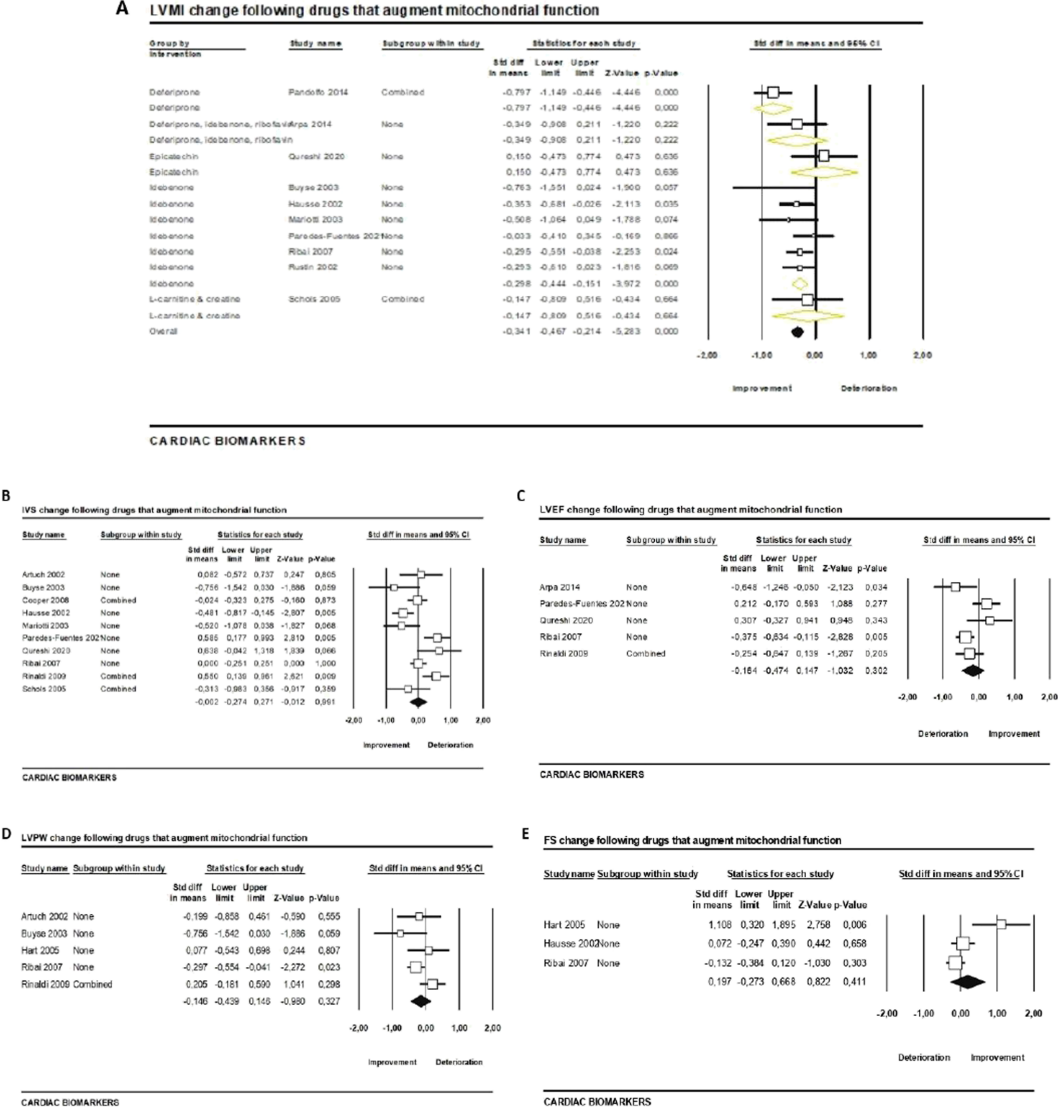
Cardiac biomarker changes following drugs that augment mitochondrial function. **A** Left ventricular mass index (LVMI), **B** intraventricular septal wall (IVS), **C** left ventricular ejection fraction (LVEF), **D** left ventricular posterior wall (LVPW), **E** fractional shortening (FS)

Left ventricular mass index (LVMI) was improved significantly (SMD =  − 0.34, 95% CI − 0.5 to 0.18, *p* < 0.001, *I*^2^ = 33%) following 28.5 months (median; range 6 to 132 months) of treatment with drugs that augment mitochondrial function. This result was based on the pooled analysis of ten studies of which eight had high risk of bias. Interestingly, the statistically significant result remained at the subgroup analysis according to the type of drug administered (SMD =  − 0.34, 95% CI − 0.47 to 0.21, *p* < 0.001, *I*^2^ = 33%, Fig. 4A). The remaining parameters assessed (IVS: SMD =  − 0.002, 95% CI − 0.27 to 0.27, *p* = 0.99, *I*^2^ = 73%; LVEF: SMD =  − 0.16, 95% CI − 0.47 to 0.15, *p* = 0.3, *I*^2^ = 63%; LVPW: SMD =  − 0.15, 95% CI − 0.44 to 0.15, *p* = 0.3, *I*^2^ = 45%; FS: SMD = 0.2, 95% CI − 0.27 to 0.67, *p* = 0.4, *I*^2^ = 77%) did not change after treatment as presented in Fig. 4B, C, D, and E, respectively.

#### Drugs that Increase Frataxin

Only four studies assessed the effect of drugs that increase frataxin on cardiac biomarkers (Table 3). Thus, we performed a pooled analysis of three studies reporting on LVEF changes [53, 56, 58].We found that LVEF remained stable during 10.5 months (range 3–18 months) of treatment with epoetin alfa, resveratrol, or IFNγ-1b (SMD =  − 0.13, 95% CI − 0.44 to 0.18, *p* = 0.4, *I*^2^ = 26%).

#### Symptomatic Treatment

There were no sufficient data presented at the three studies of symptomatic treatment assessing cardiac biomarkers to perform a quantitative synthesis (Table 3) [60, 61, 63].

### Biochemical Biomarkers

Descriptive characteristics of 17 studies that investigated biochemical biomarkers are presented in Table 4. We were not able to perform a pooled synthesis of biochemical biomarkers after treatment with drugs that augment mitochondrial function [30, 35, 46, 47, 52, 64] nor after symptomatic treatment due to the diverse biochemical biomarkers assessed.

**Table 4:** Biochemical biomarkers examined in each study included in the qualitative synthesis

ID	Drug	Biomarker	*N*	Follow-up*	Change after treatment
Drugs that augment mitochondrial function					
Buyse 2003	Idebenone	Erythrocyte protoporphyrin IX	8	12	Reduced
NINDS cohort	Idebenone	Urinary 8OH2ʹdG	48	6	No change
Pineda 2008	Idebenone	Antioxidants^+^, plasma malondialdehyde	24	60	No change
Qureshi 2020	Epicatechin	Mitochondrial FXN, urine F2-isoprostane, follistatin, myostatin	10	6	Follistatin levels increased significantly
Schulz 2000	Idebenone	Urinary 8OH2ʹdG, plasma DHBA	8	2	Urinary 8OH2ʹdG levels decreased significantly
Drugs that increase frataxin					
Innsbruck cohort	RhuEPO	FXN levels in isolated lymphocytes, urinary 8OH2ʹdG, serum peroxides	8	6	Frataxin levels increased; urinary 8OH2ʹdG and peroxide levels decreased (*p* < 0.05)
Libri 2014	Nicotinamide	FXN mRNA expression, FXN concentration	10	2	Significant upregulation of FXN expression and concentration
Lynch 2019	IFNγ-1b	FXN levels in whole blood, muscle biopsies, and buccal cells	92	6	No change
Mariotti 2012	RhuEPO	FXN in peripheral lymphocytes	16	6	No change
Austrian cohort	RhuEPO	FXN in PBMCs and skeletal muscle, NADH/NAD ratio	11	2	FXN levels and decrease NADH/NAD ratio increased significantly
Saccà 2011	EPO	PBMC FXN levels	10	15	PBMC FXN levels increased significantly
Saccà 2016	EPO	PBMC FXN levels	56	12	No change
Seyer 2014	IFNγ-1b	FXN levels in PBMCs and multiple tissues, FXN mRNA levels	12	3	Significant changes in FXN levels in red blood cells (increased), whole blood (decreased), and platelets (decreased)
IRCCS cohort	IFNγ-1b	PBMCs FXN levels	12	12	No change
Yiu 2015	Resveratrol	PBMCs FXN levels, PMBCs FXN mRNA, plasma F2-isoprostane, and urinary 8OH2ʹdG	24	3	Plasma F2-isoprostane decreased significantly
Symptomatic treatment					
Costantini 2016	Thiamine	FXN mRNA levels	34	24	Increased
Patel 2019	Methylprednisolone	Whole blood and buccal cells FXN levels	11	6	No change

*FXN* frataxin, *8OH2ʹdG* 8-hydroxy-2ʹ-deoxyguanosine, *DHBA* dihydroxybenzoic acid, *PBMCs* peripheral blood mononuclear cells

*Maximum follow-up period presented in months

^+^Tocopherol, retinol, coenzyme Q10, selenium, zinc, antioxidant enzymes in erythrocytes (superoxide dismutase, catalase, glutathione peroxidase, and glutathione reductase

#### Drugs that Increase Frataxin

**Fig. 5:**
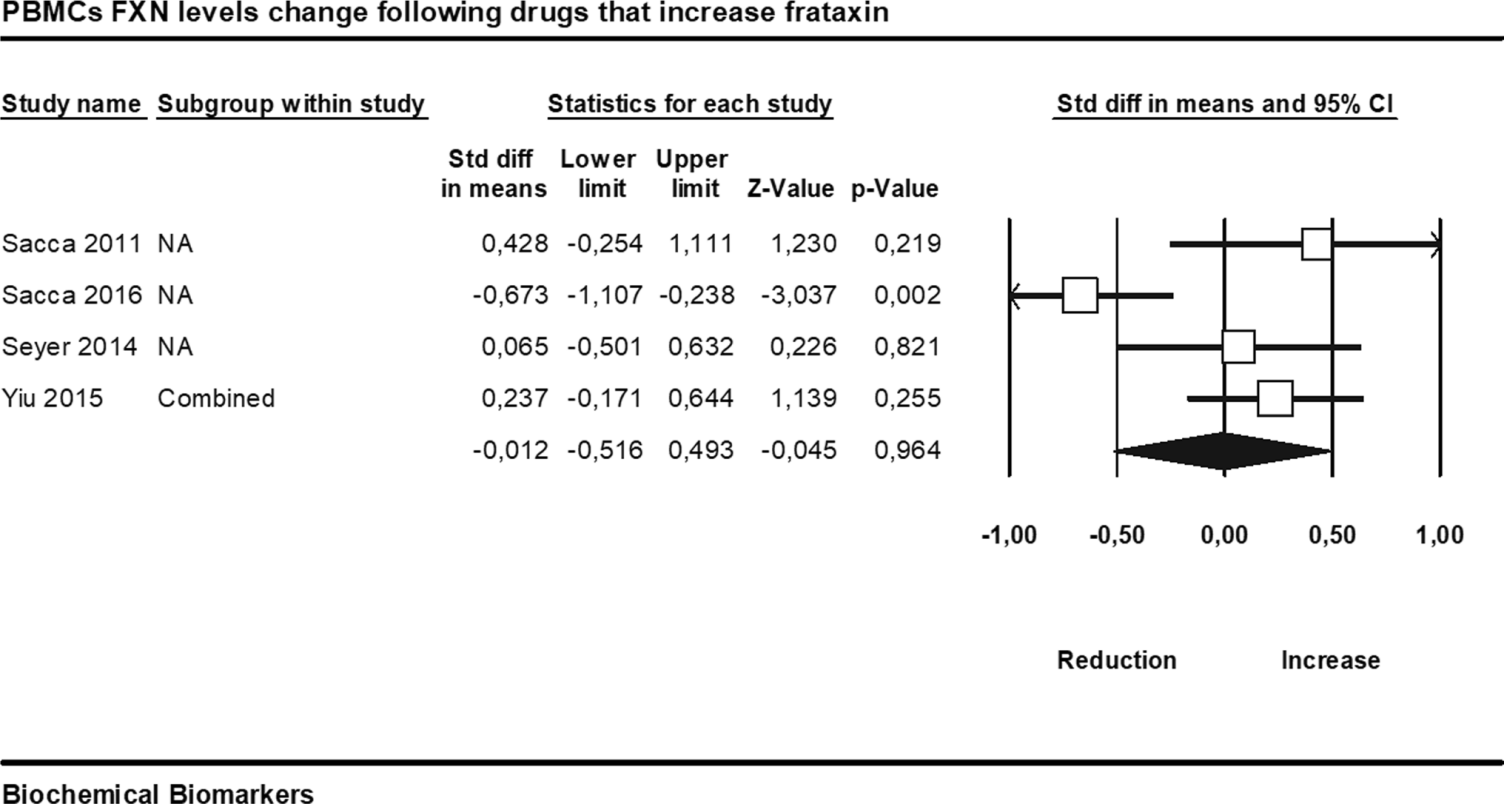
Changes in peripheral blood mononuclear cells’ (PBMCs) frataxin (FXN) levels following drugs that increase frataxin

We found no significant difference between pre- and post-treatment (median follow-up period of 3 months) with drugs that increase frataxin on peripheral blood mononuclear cells’ frataxin levels based on the pooled effect estimates of four studies (SMD =  − 0.01, 95% CI − 0.52 to 0.49, *p* = 0.96, *I*^2^ = 74%, Fig. 5).

### Patient-Reported Outcome Measures

PROMs were assessed in 21 studies as presented in Table 5.

**Table 5:** Patient-reported outcome measures (PROMs) examined in each study included in the qualitative synthesis

ID	Drug	Biomarker	*N*	Follow-up*	Patient perspective after treatment
Drugs that augment mitochondrial function					
Arpa 2014	Deferiprone, idebenone, riboflavin	SF-36v2	13	45	Dissatisfied
Brandsema 2010	Idebenone	PedsQL, ADLs	7	12	Total PedsQL and ADLS improved; physical component deteriorated
Cook 2019	Idebenone	Status and change questionnaires, MFIS	29	2	No worsening
UCL cohort [32]	Q10 and vitamin E	ADLs	59	24	Deterioration
NINDS cohort [35]	Idebenone	ADLs	48	6	No difference between placebo and treatment groups
Elincx-benizri 2015	Deferiprone and idebenone	SF-36	5	24	Deterioration
IONIA study [41]	Idebenone	ADLs	70	6	No difference between placebo and treatment groups
MOXIe Study [8, 39]	Omaveloxolone	SF-36v2, PGIC, ADLs	103	12	Improvement^+^
Pandolfo 2014	Deferiprone	ADLs	72	6	Deterioration
Qureshi 2020	Epicatechin	ADLs	10	6	Deterioration
Drugs that increase frataxin					
Innsbruck cohort [56]	RhuEPO	SF-36	8	6	Improvement^+^ in mental component; physical component did not change
Libri 2014	Nicotinamide	ADLs	10	2	Improvement
Lynch 2019	IFNγ-1b	ADLs, MFIS, PedsQL, or SF-36	92	6	No difference between placebo and treatment group
Mariotti 2012	RhuEPO	SF-36	16	6	No change
Saccà 2016	Epoetin alfa	EQ-5D, ADLs	56	12	No change
Seyer 2014	IFNγ-1b	ADLs, MFIS, PedsQL	12	3	No change
Yiu 2015	Resveratrol	FAIS, SF-36v2	24	3	No change
Symptomatic treatment					
Costantini 2016	Thiamine	FSS	34	24	No change
Patel 2019	Methylprednisolone	ADLs, MFIS, SF-36, PGI	11	6	No change
Sanz-Gallego 2014	IGF-1	SF-36v2	5	36	Satisfied
Wang 2021	Luvadaxistat	ADLs, PGI	67	3	No change

*SF-36v2* Short Form Health Survey version-2.0, *PedsQL* Pediatric Quality of Life Inventory, *ADLS* Activities of Daily Living Scale, *MFIS* Modified Fatigue Impact Scale, *PGIC* Patient Global Impression of Change, *FSS* Fatigue Severity Scale, *FAIS* Friedreich Ataxia Impact Scale

*Maximum follow-up period presented in months

^+^Statistically significant

#### Drugs that Augment Mitochondrial Function

**Fig. 6:**
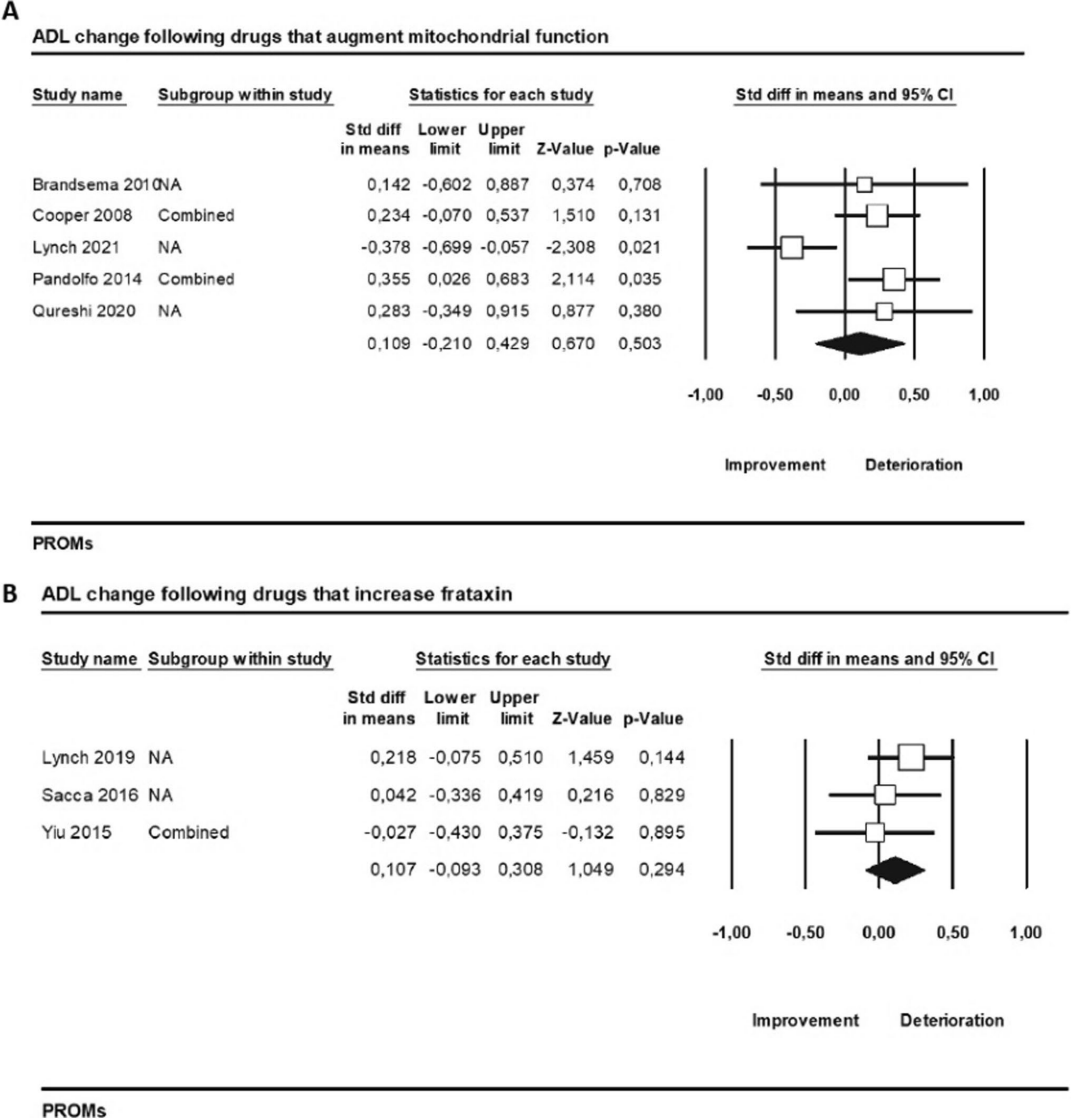
Activities of Daily Living Scale (ADLs) changes following **A** drugs that augment mitochondrial function, **B** drugs that increase frataxin

We found that ADLs remained stable (SMD = 0.11, 95% CI − 0.21 to 0.43, *p* = 0.5, *I*^2^ = 66%) after 12 months (median follow-up) of treatment with drugs that augment mitochondrial function according to five studies (Fig. 6A).

#### Drugs that Increase Frataxin

ADLs were also not affected by drugs that increase frataxin according to the pooled effect of three studies (SMD = 0.11, 95% CI − 0.09 to 0.31, *p* = 0.3, *I*^2^ = 0%) presented in Fig. 6B.

#### Symptomatic Treatment

The four studies examining symptomatic approaches revealed no treatment effect on PROMs based on the qualitative synthesis (Table 5).

### Imaging Biomarkers

Imaging biomarkers were assessed in nine studies (11 records); five studies examined the effect of drugs that augment mitochondrial function on imaging parameters [28, 33, 34, 47, 51, 54], one investigated a symptomatic treatment [75] while the intervention of the remaining three studies was aiming to increase frataxin [57, 58, 65, 70].

#### Drugs that Augment Mitochondrial Function

Boddaert et al. showed that a 6-month deferiprone treatment led to reduction of iron accumulation specifically in dentate nuclei by performing brain magnetic resonance imaging (MRI) at FRDA patients [28]. The same finding was confirmed by Velasco-Sánchez et al. after 11 months of combined deferiprone and idebenone treatment [54].

Cardiac and skeletal muscle phosphorus P31 magnetic resonance spectroscopy (MRS) was used by the UCL study group [33, 34]. A significant amelioration of cardiac and skeletal muscle bioenergetics was found following combined Q10 and vitamin E treatment. P31 MRS was also examined by Schöls et al. [51]. l-Carnitine phosphocreatine recovery was improved following 4 months of l-carnitine and creatine treatment. However, no difference was found compared to placebo group.

Recently, Qureshi et al. used a variety of imaging parameters such as spinal cord and cerebellar volume measured by 3D volumetric MRI, spinal cord fractional anisotropy by diffusion tension imaging (DTI), cerebellar metabolite ratios by 3D MRS along with cardiac MRI to monitor epicatechin administration [47]. A significant reduction of LV mass index at cardiac MRI was reported. Moreover, Qureshi et al. found a significant reduction at mean cerebellar volume but without subsequent worsening among individual patients after 24 weeks.

#### Drugs that Increase Frataxin

Recombinant human erythropoietin effect on imaging biomarkers was assessed by two studies presented at three records [57, 58, 65]. Axial diffusivity changes were detected in cerebral hemispheres by DTI, but this finding did not correlate with any clinical outcome [58]. Santner et al. found an increase of pulvinar and the posterior parietal cortex gray matter volume after 6-month treatment with rhuEPO using voxel-based morphometry [57]. Interestingly, this observation correlated with an improvement in clinical scores. P31 MRS examination of the calf muscles did not change following 2 months of rhuEPO administration in a study by Nachbauer et al. [65].

Valva et al. reported interferon gamma treatment induced changes on advanced MRI and retinal imaging [DTI, functional MRI (fMRI), resting-state fMRI (rs-fMRI)]. Significant alterations were detected on fMRI and rs-fMRI; the former correlated with clinical outcomes [70].

#### Symptomatic Treatment

Two studies assessed the effect of idebenone, a drug which augments mitochondrial function, on neurophysiologic biomarkers. Naeije et al. performed a sham-controlled, crossover RCT using anodal cerebellar transcranial direct current stimulation (ctDCS) in 24 FRDA patients with a follow-up period of 3 months [75]. A reduced cSII cortex functional magnetic resonance imaging (fMRI) response was elicited by a tactile oddball stimulation following ctDCS compared with sham ctDCS probably because of the restoration of the neocortical inhibition normally exerted by the cerebellum.

### Neurophysiologic and other Biomarkers

Two studies assessed the effect of idebenone, a drug which augments mitochondrial function, on neurophysiologic biomarkers [27, 53]. Electromyography, somatosensory, and visual evoked potentials parameters did not change following 12 or 24 months of treatment. However, peroneal motor nerve conduction velocity deteriorated significantly as reported by Sival et al. [53].

Other biomarkers such as exercise testing and muscle biopsy were examined by four studies (presented in five records) [8, 36, 39, 64, 66]. Idebenone or epoetin alfa treatment did not affect peak oxygen consumption per unit time or peak work rate according to Drinkard and Saccà et al., respectively [36, 66]. However, a nonsignificant improvement in peak work was observed by Lynch et al. at the MOXIe Study [8, 39]. Muscle tissue changes after administration of recombinant human erythropoietin were investigated by Nachbauer et al. [64]. FRDA patients showed reduced respiratory chain complex and citrate synthase activities in skeletal muscle compared with healthy controls but were not affected by treatment.

### Biomarker Change over Time in No Treatment Group

We performed an exploratory analysis using data of biomarker trajectory over time in the untreated patient group to elucidate the ability of a biomarker to detect subtle disease progression. We found that none of the biomarkers examined (ADLs, FARS, ICARS, LVMI, mFARS, T25FW^−1^, 9HPT^−1^) changed significantly over a median follow-up period of 6 months. Data of the quantitative analyses are summarized in Table 6.

**Table 6:** Pooled analyses data summary of biomarkers changes over time in untreated patients

Outcome	No. of studies	Follow-up [median (range)], months	SMD (95% CI), *I*^2^
ADLs	5	6 (3–12)	0.46 (− 0.31 to 1.23), 93%
FARS	3	6 (6–24)	− 0.13 (− 0.36 to 0.09), 0%
ICARS	4	6 (2–60)	0.12 (− 0.44 to 0.68), 48%
LVMI	5	12 (6–60)	0.05 (− 0.3 to 0.41), 75%
mFARS	3	6 (3–12)	0.05 (− 1.14 to 1.25), 97%
T25FW^−1^	3	6 (6–12)	− 1.42 (− 3.03 to 0.18), 97%
9HPT^−1^	3	6 (3–12)	− 0.04 (− 0.25 to 0.17), 0%

*ADLs* Activities of Daily Living Scale, *LVMI* left ventricular mass index, *FARS* Friedreich Ataxia Rating Scale, *T25FW* Timed 25-Foot Walk, *9-HPT* 9-hole peg test, *ICARS* International Cooperative Ataxia Rating Scale, *SMD* standardized mean difference

### Certainty of Evidence

We applied Grading of Recommendations Assessment, Development and Evaluation (GRADE) tool to assess quality of evidence of our estimates which was low to very low due to the observational nature, the high RoB, along with the inconsistency of included studies (online-only Supplementary material 4).

## Discussion

### Summary of Evidence and Implications for Practice

The present meta-analysis explored the effect of different types of interventions targeting mitochondrial function, frataxin, or patients’ symptoms on clinical, cardiac, biochemical, PROMs, imaging, or neurophysiologic biomarkers in 1409 patients with Friedreich ataxia. In the context of the 43 included studies, a large array of biomarkers was applied as outcome measures. A statistically significant improvement was detected in Friedreich Ataxia Rating Scale scores (combining FARS and mFARS as clinical outcome measures) in 205 patients after 15 months of treatment with drugs that augment mitochondrial function. Nevertheless, this result should be interpreted with caution because it was mainly driven by omaveloxolone’s positive trial and was characterized by very low quality of evidence. Low quality of evidence from ten studies (seven of which were observational) examining 261 patients supported a beneficial effect of drugs that augment mitochondrial function on cardiac structure measured by LVMI after 28.5 months. This result was driven by idebenone studies in combination with one trial of the iron chelator deferiprone. In contrast, all the remaining biomarkers examined did not change following any treatment or during the natural course of the disease. Of note, the median follow-up period for these outcome measures did not exceed 12-months.

An ideal valuable biomarker should be able to detect both subtle changes in the natural course of a slowly progressive disorder such as Friedreich ataxia and concurrently be responsive to any treatment effect. Nevertheless, it is imperative that the observed alterations are also clinically meaningful. The pooled analysis in untreated patients showed that LVMI did not change over 12 months. The observed LVMI reduction following drugs that augment mitochondrial function without any parallel change in other cardiac or clinical biomarkers should be interpreted with caution. In view of these results, we suggest the use of a biomarker toolbox (for example, a combination of Friedreich Ataxia Rating Scale scores and LVMI assessment) evaluating different aspects of this disease as a primary outcome measure in future RCTs. Notably, study duration should be least 24 months considering that trials with shorter duration are unlikely to demonstrate any clinical benefit. Finally, the clinical meaningful change of the employed biomarkers should be predefined based on natural history studies.

### Evidence from other Studies

A Cochrane review was conducted in 2016 to assess the therapeutic efficacy of pharmacological treatments for Friedreich ataxia [80]. Hence, only RCTs with a minimum follow-up of 12 months were included in this Cochrane review. Furthermore, Jain et al. carried out a systematic literature review (published at 2022 before the FDA approval of omaveloxolone) to summarize the efficacy and safety of therapeutic interventions that have been investigated in Friedreich’s ataxia [81]. On the contrary, we aimed to explore the effect of the interventions on biomarkers in order to assess their response to change. Thus, we did not limit our search to RCTs and set a minimum 2-month interval between biomarker assessments that resulted in the exclusion of some records from the present meta-analysis [82–84]. We found no change at any biomarker examined either in treated or untreated patients except for a statistically significantly reduction in Friedreich Ataxia Rating Scale scores (combining FARS and mFARS) and LVMI following drugs that augment mitochondrial function. In line with our findings, Jain et al. conclude that the limited sample size and follow-up duration led to inconclusive evidence. Similarly, LVMI was only investigated by one RCT included in the Cochrane review in which a significant decrease was detected [80]. Nevertheless, the clinical relevance of this result was interpreted with caution in the Cochrane review due to the low quality of evidence in line with our conclusions.

We limited this review to studies examining a therapeutic intervention but also investigated the change from baseline score of any biomarker in untreated patients separately. The EFACTS study group evaluated 552 treatment-naive patients exclusively with at least 4 years of follow-up in a prospective cohort study and found an annual progression rate of 0.82 points (SE 0.05) for SARA and 0.93 points (SE 0.05) for ADL [85]. They calculated that 190 patients would be required to detect a 50% ADL reduction in a 2-year parallel-group trial. We found a non-significant ADL SMD change of 0.46 (− 0.31 to 1.23) in 147 patients followed for a median period of 6 months. The observed difference could be attributed to the limited follow-up time of the studies included in this review.

## Strengths and Limitations

The observational nature of the majority of the included records in conjunction with the lack of a control group, the short median follow-up duration in most outcomes, and the heterogeneity of the examined populations represent the major limitations of this meta-analysis. Thus, we downgraded the quality of evidence of our estimates. Management of FRDA requires a multidisciplinary approach consisting of numerous interventions (such as occupational and physical therapy, speech and swallowing therapy, psychological counseling) which could not be pooled in one systematic review. Accordingly, we conducted a comprehensive literature search following PRISMA guidelines focusing on pharmacological therapies along with non-invasive neurostimulation approach, in line with our prespecified protocol. This led to the inclusion of 1409 patients in the current meta-analysis, a large population cohort considering that Friedreich ataxia is a rare disease.

## Conclusions and Future Perspectives

A statistically significant improvement was detected in Friedreich Ataxia Rating Scale scores [combining Friedreich Ataxia Rating Scale (FARS) and modified FARS (mFARS), SMD =  − 0.32 (− 0.62 to − 0.02)] in 205 patients following 15 months of treatment drugs that augment mitochondrial function in a sensitivity analysis of six studies with very low quality of evidence. Moreover, low-quality evidence from ten, mainly observational, studies evaluating 261 patients suggested a significant reduction of LVMI after a median 28.5 months of treatment with drugs that augment mitochondrial function. The clinical importance of these changes remains to be clarified in well-designed RCTs in which the minimal clinically important change should be predefined. We found no significant change on any other clinical, cardiac, biochemical, PROMs, imaging, or neurophysiologic biomarker investigated in 1409 patients with Friedreich ataxia. Nevertheless, the median follow-up period for these outcomes was extremely limited considering the slow natural disease progression. Hence, a biomarker toolbox evaluating different aspects of this complex disease (for example, combining both Friedreich Ataxia Rating Scale scores and LVMI assessment) could be applied as a primary outcome measure in future RCTs of at least 24 months’ duration.

### Supplementary Information

Below is the link to the electronic supplementary material.

Supplementary file1 (DOCX 167 KB)

## Data Availability

All data underlying this study are available in this article and in its online supplementary material.
